# Germline Genetic Modification and Identity: the Mitochondrial and Nuclear Genomes

**DOI:** 10.1093/ojls/gqx012

**Published:** 2017-08-11

**Authors:** Rosamund Scott, Stephen Wilkinson

**Keywords:** CRISPR/Cas-9, genetic modification, genome editing, germline, identity, mitochondrial replacement

## Abstract

In a legal ‘first’, the UK removed a prohibition against modifying embryos in human reproduction, to enable mitochondrial replacement techniques (MRTs), a move the Government distanced from ‘germline genetic modification’, which it aligned with modifying the nuclear genome. This paper (1) analyzes the uses and meanings of this term in UK/US legal and policy debates; and (2) evaluates related ethical concerns about identity. It shows that, with respect to identity, MRTs and nuclear genome editing techniques such as CRISPR/Cas-9 (now a policy topic), are not as different as has been supposed. While it does not follow that the two should be treated exactly alike, one of the central reasons offered for treating MRTs more permissively than nuclear genetic modification, and for not regarding MRTs as ‘germline genetic modification’, is thereby in doubt. Identity cannot, by itself, do the work thus far assigned to it, explicitly or otherwise, in law and policy.

## 1. Introduction

When, in 2015, the UK Parliament passed regulations permitting the use of mitochondrial replacement techniques (MRTs) to prevent the transmission of serious mitochondrial disease,^^1^^ a long-standing prohibition against modifying embryos in the course of in vitro fertilisation (IVF) was removed for the first time in the world, accompanied by recognition of the heritability of such modifications.^2^ The Human Fertilisation and Embryology (Mitochondrial Donation) Regulations 2015 (the Regulations) were against the backdrop of various international statements and conventions which take a cautious or prohibitive stance towards germline genetic intervention, as well as widespread international prohibition.^3^ While several concerns, notably regarding risk,^4^ underlie these cautious or prohibitive approaches, a fundamental one is the supposed wrongness of altering the ‘identities’ of future people. Perhaps not surprisingly then, but nonetheless somewhat curiously, the UK Government emphasized that while MRTs ‘do result in *germ-line* modification … the techniques [do not] constitute *genetic* modification’.^5^ Subsequently, early in 2016, a US Food and Drug Administration (FDA) commissioned report of an *ad hoc* committee of the US Institute of Medicine (IOM) held that MRTs *do* constitute genetic modification and that, since mitochondria are maternally inherited, MRTs *also* amount to germline modification if female offspring are born. Despite this strikingly different conclusion, the report adopted a cautiously permissive approach to MRTs (although a section in a federal statute passed shortly before publication of the IOM Report, and subject to annual renewal, currently bars an FDA decision on the safety and efficacy of MRTs).^6^

Given the significance of the legal developments in the UK and the prospect of the US and other countries moving permissively to regulate MRTs,^7^ this paper has two aims: *first*, to analyze the uses and meanings of ‘germline genetic modification’ in the UK and US legal and policy debates; *second*, to evaluate ethical concerns about ‘identity’ raised by MRTs. The analysis has implications for policy debates regarding the reproductive use of nuclear genome editing techniques, such as CRISPR/Cas-9,^8^ that have now begun in earnest.^9^

*Section 2* situates the legalisation of MRTs within the UK legal framework. It also considers the position on germline genetic modification in several key international statements and conventions and how the revised UK legal position stands in relation to these.

*Section 3* explores possible justifications for the view that MRTs do not constitute germline genetic modification with reference to UK and US policy, regulatory, legal, scientific and academic materials. The UK classification of MRTs as something other than germline genetic modification involved construing the concept more *narrowly* than might otherwise be the case; two key distinctions were in play in this ‘narrowing’. First, and principally, MRTs directly concern only the *mitochondrial*, not the nuclear genome. Second, MRTs involve *replacement* (of one whole ‘naturally occurring’ mitochondrial genome with another), not *modification.* The supposed normative significance of these distinctions is that, compared to nuclear genome editing, MRTs: (a) are unlikely to alter in significant ways the identity of the person created; (b) do not introduce ‘artificial’ elements into the gene pool; and (c) are less likely to be used for human enhancement. These points played a key role in justifying the UK legalisation of MRTs. Although the US IOM report reached different conclusions about germline genetic modification, the same distinctions underpin its cautiously permissive approach.

Finally, *Section 4* critically assesses the argument that modifying the nuclear genome is more ethically troubling than MRTs because that could, to a greater extent, affect the ‘identities’ of future people. The section’s main conclusion is that, while there is no categorical difference (regarding identity) between MRTs and modifying the nuclear genome, a more precautionary approach to the latter may be justified because of its greater potential for non-therapeutic use.

Overall, the analysis shows that the distinction between mitochondrial and nuclear and that between replacement and modification, coupled with the appeal to identity, cannot do the work typically placed on them. Rather, the permissibility of an intervention in either the mitochondrial or nuclear genome depends on the context, extent and nature of the intervention in question.

## 2. MRTs and the UK Legal and International Background

We first consider recent changes to the UK legal position, the nature of the proposed MRTs, and where the UK stands in relation to the international legal position.

### A. UK Legal Background

The Human Fertilisation and Embryology (HFE) Act 1990 established a world-leading legal and regulatory framework for assisted reproduction and embryo research. Over time, however, scientific developments prompted the courts and Parliament to respond in various ways. For instance, the development of somatic cell nuclear transfer (SCNT) led to a legal scare regarding the scope of the HFE Act to govern the use of embryos created by means other than fertilisation, entailing both a legal challenge to the Act^10^ and emergency legislation (the Human Reproductive Cloning Act 2001). Against this background, the 2008 revisions to the HFE Act made explicit that it applies to *all* human embryos, no matter how created.^11^ The Act now excludes from reproductive use embryos not created by fertilisation, a task previously fulfilled by means of the 2001 Act. It distinguishes ‘permitted’ from other embryos (and gametes) such that only ‘permitted’ ones may be used in treatment.^12^ MRTs have been accommodated within this framework.

The HFE Act was amended in 2008 such that regulations could provide that eggs or embryos would be ‘permitted … even though the egg or embryo has had applied to it in prescribed circumstances a prescribed process designed to prevent the transmission of serious mitochondrial disease’.^13^ Two ‘processes’ have been the subject of research, at least one of which may shortly be used in treatment in the UK - maternal spindle transfer (MST) and pronculear transfer (PNT):

*Maternal spindle transfer (MST).* The ‘maternal spindle’ is the group of maternal chromosomes within the egg, which are shaped in a spindle. MST involves removing the spindle from the mother’s egg before it is fertilised by the father’s sperm. The spindle is then placed into a donor egg with healthy mitochondria (from which the donor’s spindle, and therefore her nuclear material, has been removed).

*Pro-nuclear transfer (PNT).* The pro-nucleus is the nucleus of a sperm or an egg cell during the process of fertilisation after the sperm enters the egg, but before they fuse. PNT involves removing the pro-nuclei (nuclear material) from a newly fertilised egg (which is regarded as an embryo under the Human Fertilisation and Embryology Act 1990) that has unhealthy mitochondria. The pro-nuclei are then transferred into a donated embryo, with healthy mitochondria, that has had its own, original pro-nuclei removed.^14^

The subsequently passed Regulations establish that ‘permitted’ eggs or embryos can have been the subject of particular specified processes (which detail the relevant methods) in specified circumstances (which concern the risk of disease).^15^ The Regulations also provide that there must have been ‘no alterations in the nuclear or mitochondrial DNA’ either of an egg following MST, or of an embryo following PNT.^16^

We next consider how these techniques should be viewed with reference to the concept of ‘germline genetic modification’, looking first at key international statements and conventions.

### B. International Statements and Conventions

The UK Government’s position that PNT and MST are *not* forms of ‘germline genetic modification’ is influenced by long-standing international opposition to such modification (especially of human beings).^17^ Consider Sir Edward Leigh’s contribution to the House of Commons debate in which he asked whether ‘we really want to become a rogue state in terms of bioethics?’.^18^ It is therefore instructive to see how the UK’s legalisation of MRTs fits into this international context.

By way of introduction, we note three points. First, international statements and conventions use a range of terms relevant to the discussion of germline genetic modification and these require interpretation. Second, the implications for the permissibility of particular practices are sometimes unclear. Third, at least in one early statement, there are indications of factors which might support the permissibility of germline modifications in particular circumstances, such as where the aim is to treat or eradicate disease.

This early statement is the Parliamentary Assembly of the Council of Europe’s 1982 recommendation on *Genetic Engineering.* Paragraph 4(a) boldly asserts that ‘the rights to life and to human dignity protected by Articles 2 and 3 of the European Convention on Human Rights [ECHR] imply the *right to inherit a genetic pattern which has not been artificially changed*’.^19^ Whether, or in what way, MRTs involve such changes is, as discussed below, an important issue. For now, we note that this statement relies on a considerable degree of interpretation of those articles, respectively the right to life and the right (in part) not to be subjected to ‘inhuman or degrading treatment’.^20^ Paragraph 4(b) adds that ‘this right should be made explicit in the context of the European Convention on Human Rights’. However, as of 2017 – 35 years later – the European Court of Human Rights (ECtHR) has not yet done so. In any event, paragraph 4(c) shifts the tone, holding:

[T]he explicit recognition of … [the right to inherit a genetic pattern which has not been artificially changed] must not impede development of the *therapeutic applications of genetic engineering (gene therapy)*, which holds great promise for the *treatment* and *eradication* of certain diseases which are *genetically transmitted.*^21^

This strikes a more permissive note regarding ‘gene therapy’ although the meaning of that expression is unclear. It seems unlikely that it refers only to ‘somatic’ gene therapy (therapy relating to a cell in the human body that is not a germ cell)^22^ as this cannot *eradicate* genetically inherited disease. Furthermore, the notion of ‘eradication’, together with the phrase ‘genetically transmitted’ and paragraph 4(c)’s implicit reference back to paragraph 4(a)’s statement regarding inheritance, suggests a potentially permissive (though guarded) approach towards germline modification.

In this light, MRTs may be consistent with paragraph 4(c) if they treat an individual or eradicate disease, a framing prevalent in the UK policy debate.^23^ Since under both the amended and original HFE Act, the HFEA can only issue licences for research, treatment, or storage,^24^ when MRTs move out of the research setting, they will necessarily be classed as ‘treatment’ for regulatory purposes. Whether however MRTs should be viewed as treatment in a wider sense (that is, for purposes other than regulatory classification) is a more complex and controversial question. PNT may be seen as ‘treatment’, because it occurs after the development of the embryonic pronuclei, and thus is something that happens to a determinate individual.^25^ By contrast, MST may be viewed as selective reproduction, because mitochondrial replacement occurs before fertilisation.^26^ These issues are discussed in *Section 4.*

It is also worth noting here that whether an intervention is a ‘treatment’ in a wider sense, or in an ethical or philosophical sense, may be (at most) only very indirectly related to its *regulatory* status. For example, in the context of avoiding the birth of a child with a serious genetic condition, treatment licenses have previously been granted for techniques which do not treat the future child. Under the original Act (as a matter of statutory interpretation)^27^ and now the amended Act, treatment licences have been granted for preimplantation genetic diagnosis (PGD), a form of selection described as ‘testing embryos’ and situated as occurring ‘in the course of providing treatment services’,^28^ themselves defined in the Act as ‘medical, surgical or obstetric services provided to the public or a section of the public for the purpose of assisting *women* to carry children’.^29^ At the same time, with their various references to ‘therapy’, the consultation and policy materials leading to the passage of the MRT Regulations cast MRTs as ‘treatment’ of a *future person.*^30^ In relation to MRTs, there is thus some *fluidity* in the interpretation of ‘treatment’ in the UK policy and regulatory context, which likely has a normative purpose in the debate.

In contrast to the UK’s characterisation of MRTs as ‘treatment’ of a future person, the US IOM report emphasizes that MRTs do *not* treat and that the concept of ‘treatment’ can only be understood in this context as treatment of the prospective parents.^31^ The IOM report also stresses that MRTs do not prevent disease in existing people,^32^ which is consistent with the UK policy position.

Regardless of whether MRTs *treat* disease, they may nonetheless contribute to the *eradication* of disease, and so could be compatible with the Council of Europe’s 1982 Recommendation on that basis. In this light, even if Articles 2 and 3 of the ECHR could reasonably be interpreted, as the Council recommended, to ‘imply the right to inherit a genetic pattern which has not been artificially changed’ (which is doubtful), it is questionable whether this could (or should) include the right to inherit disease. In any event, the meanings and significance of ‘genetic’ and of ‘artificially changed’ are contested, as *Section 3* (below) shows.

The vagueness of the 1982 Recommendation’s references to ‘respect for human rights’ was criticized, the same year, in a guardedly permissive US President’s Commission Report, *Splicing Life*,^33^ which warned of the ‘risk … of depriving humanity of the great benefits genetic engineering may bring’.^34^ Yet, since the early 1980s, various developments have increased concern about germline genetic modification. These include the publication of the majority of the human genome in 2001,^35^ as well as the development of SCNT, which triggered alarm and the widespread prohibition of human reproductive cloning.^36^

Perhaps it is not surprising therefore that a 2003 report of the International Bioethics Committee (IBC) of the United Nations Educational, Scientific and Cultural Organization (UNESCO) found legislation in nine countries, including the UK, apparently prohibiting germline modification (using various expressions).^37^ The previous year a World Health Organisation (WHO) Bulletin piece stated that ‘most [relevant] ethical and legal regulations that cover this issue strongly discourage or frankly prohibit [germline interventions]’.^38^ The international statements and conventions in question are very significant, but require interpretation with reference to MRTs.

First, the 1997 UNESCO Universal Declaration on the Human Genome and Human Rights holds, in Article 1, that ‘[t]he human genome underlies the fundamental unity of all members of the human family, as well as the recognition of their inherent dignity and diversity. In a symbolic sense, it is the heritage of humanity’.^39^ Article 5(a) states (in part) that ‘[r]esearch, *treatment* or diagnosis *affecting an individual’s genome* shall be undertaken only after rigorous and prior assessment of the potential risks and benefits’, as well as reference to relevant national laws.^40^ Whether the latter emphasized passage refers only to somatic or also to germline interventions is not clear. Article 5(b) stresses the importance, ‘[i]n all cases’ of ‘the prior, free and informed consent of the person’, observing that if this is not possible, ‘consent or authorization’ should be guided by ‘best interests’, with due regard to relevant national laws. This suggests a focus on somatic interventions. Indeed, looking more broadly at the Convention, Article 11 reads: ‘Practices which are contrary to human dignity, such as reproductive cloning of human beings, shall not be permitted’. More particularly, Article 24 refers to the IBC (of UNESCO) making recommendations regarding ‘the identification of practices that *could be* contrary to human dignity, such as *germ-line interventions*’.^41^ This suggests that Article 5 is best interpreted as referring only to *somatic* interventions. It certainly implies that, under the Convention, techniques such as MRTs need to be considered with reference to the concept of dignity. Taking on this challenge, the UK Government confidently stated:

In bringing forward regulations to enable mitochondrial donation we have been mindful of the UK’s obligations under international law. We do not consider that permitting mitochondrial donation, *aimed at preventing serious hereditary conditions*, would be contrary to human dignity as envisaged by Article 24 of the UNESCO declaration.^42^

The Government’s stress on the preventative purpose of MRTs highlights their positive aims and comparatively limited scope for effecting genetic change. It also suggests that treating disease does not threaten dignity, at least where issues of consent or best interests (for those who lack capacity) are addressed.^43^ However, after the change to the UK legal position to accommodate MRTs, and following developments relating to genome editing in the latter half of 2015, the IBC called for ‘a moratorium on genome editing of the human germline’, at least while safety concerns prevail; further, with reference to the development of MRTs, it noted the 2012 recommendation of the Nuffield Council on Bioethics (NCoB) that MRTs should be ‘adequately proven to be acceptably safe and effective as treatments’ before entering clinical practice, but suggested that ‘even’ scientists disagree on the fulfilment of this standard and that ‘the debate remains open on the *ethical* acceptability of [MRTs]’.^44^ As was the UK Government, however, the IOM was doubtful that the UNESCO declaration should stand in the way of MRTs.^45^

Second, the Council of Europe’s 1997 Convention on Human Rights and Biomedicine (the Oviedo Convention) currently has 35 Member State signatories and 29 ratifications, and is described by the Council of Europe as a ‘framework Convention aiming at protecting dignity and identity of all human beings’.^46^ This is legally binding although the UK is *not* a signatory. Article 13, ‘Interventions on the human genome’, states that ‘an intervention seeking to *modify the human genome* may only be undertaken for *preventive, diagnostic or therapeutic purposes* and only if its *aim is not to introduce any modification in the genome of any descendants*’.^47^ The addition of the latter emphasized phrase signifies that germline modification of the ‘human genome’ is impermissible. That said, several questions remain. For example, what does ‘aim’ mean?^48^ In the UK policy debate, it was noted that the aim would be to prevent a person being born with mitochondrial disease, but *also* that future generations would benefit (by not carrying mitochondrial disease).^49^ Would the first point be caught by this article? What about the second, which could either be viewed as a secondary aim, or a foreseen effect? The terms ‘modification’ and ‘genome’ also require interpretation. Notwithstanding these uncertainties, in late 2015, the Council of Europe’s Committee on Bioethics was ‘convinced that the … Convention provides principles that could be used as reference for the debate called for at international level on the fundamental questions raised by … recent technological developments’ such as CRISPR/Cas-9 and signalled its intention to use the principles to do so itself.^50^

Compared with the Council of Europe’s 1982 Recommendation, the 1997 UNESCO declaration and the 1997 Oviedo Convention take more overtly negative stances towards germline genetic intervention, due to concerns about human dignity and identity.^51^ Indeed, the 2002 WHO Bulletin piece suggested that ‘what is at stake in [prohibiting] … human genetic engineering … is nothing less than the preservation of the identity of the human species’.^52^ Furthermore, as can be seen from the 2015 statements of the UNESCO IBC and the Council of Europe’s Committee on Bioethics, both the UNESCO declaration and the Oviedo Convention are set to play a role in debate prompted by recent developments in nuclear genome editing. So although MRTs ‘are not germ-line interventions in the sense that originally animated ethical debate’,^53^ in the light of the international context, it is understandable that the UK Government has been at pains to maintain that they are *not* ‘germline genetic modification’.

## 3. ‘Germline Modification’ and ‘Genetic Modification’: the Key Distinctions in Play

This section explores both interpretative and bioethical justifications for the UK Government position that MRTs are not ‘germline genetic modification’. It considers how ‘germline modification’ and ‘genetic modification’ have been defined and used in the UK and US debates and shows that the UK Government’s claim that MRTs are not ‘germline genetic modification’ relies on two distinctions: principally between the mitochondrial and nuclear genomes, and also between ‘replacement’ and ‘modification’. Although the US IOM report concludes that MRTs *are* germline genetic modification, reliance on these distinctions is also central to its approach. Underlying them is a fundamental concern with not controlling or altering ‘identity’, especially by ‘artificial’ means.

### A. The Mitochondrial versus the Nuclear Genome: the Major Theme

A key concern in the UK House of Commons debate was whether to permit MRTs would be to permit germline ‘genetic modification’. When questioned, the Under-Secretary of State for Health, Jane Ellison, maintained both that there is no internationally accepted definition of ‘genetic modification’ and that mitochondrial replacement is not an instance of it,^54^ in line with the *Government’s Response to the Consultation on the Draft Regulations* in 2014:

There is no universally agreed definition of ‘genetic modification’ in humans. … The working definition that we have adopted is that *genetic modification* involves the *germ-line modification* of *nuclear DNA* (in the chromosomes) that can be passed on to future generations.^55^

Whether MRTs are germline genetic modification was subsequently a key point in evidence taken by the House of Commons Science and Technology Committee (HCSTC). Professor Sally Davies, Chief Medical Officer (CMO), explained the rationale for the Government’s approach:

Germline is anything that is done to DNA that goes through the generations, and mitochondria go from woman to child through the generations. This is *clearly a germline modification because it passes through*, but we needed to make the *distinction between nuclear DNA*, which makes us *who we are and how we are –* our personalities, heights, weights and whether or not we get baldness – *and the 37 genes in the mitochondria* which are about *energy for the cell*, and which we describe as the power pack. That was why we adopted that working definition.^56^

While the CMO holds that MRTs are a ‘germline modification’, she moves swiftly to contrast the mitochondrial and nuclear genomes. The reference to ‘who we are and how we are’ implies that the nuclear genome is determinative of ‘identity’ and thus highly influential and significant. By contrast, the role of mitochondria is ‘only’ energy production. Likewise, in its consultation on the draft regulations, the DH notes that ‘it is genes in our *nuclear* DNA, together with environmental factors, rather than mitochondrial DNA, that *shape our personal characteristics and traits.*’^57^ In this light, ‘[m]ost importantly’, the DH emphasizes, ‘*mitochondrial donation* techniques *do not alter personal characteristics and traits*’.^58^

So although described by the UK Government as ‘germline modification’, MRTs are distinguished from ‘germline *genetic* modification’ on the basis that the *nuclear*, rather than the mitochondrial genome, determines ‘personal characteristics and traits’. Thus, on the Government’s view, mitochondrial replacement is not ‘germline genetic modification’ because it does not alter these aspects of a person’s *identity.* By implication, it is therefore far less significant than nuclear intervention. We consider to what extent this is justifiable in *Section 4.*

Very significantly, this association of the nuclear genome with ‘identity’ appears to have been influenced by the reasoning underlying the HFEA decision to license research into PNT at the Newcastle Fertility Centre at Life.^59^ Following an initial rejection, the application went to an HFEA Appeal Committee, which considered two key issues. First, was the proposed research prohibited by section 3(3)(d) of the original 1990 Act? This reads: ‘(3) A licence cannot authorise – … (d) replacing a nucleus of a cell of an embryo with a nucleus taken from a cell of any person, embryo or subsequent development of an embryo’? In response, the Committee noted that ‘the proposed research would involve the removal of the pronuclei at the one cell (zygote) stage’ and it ‘accepted the view that … “the zygote … at no stage contains a single nucleus …”’.^60^ Second, did Schedule 2, paragraph 3(4) of the original HFE Act prohibit the research? That paragraph reads: ‘A licence under this paragraph cannot authorise *altering the genetic structure* of any cell while it forms part of an embryo.’^61^ Critically, if the research were deemed to involve such alteration, it could not be licenced.

The Committee observed that ‘genetic structure’ was ‘ambiguous’ and therefore used various ‘interpretative criteria’.^62^ First, it ‘accepted the view of the scientific community … that when pressed to give meaning to the phrase, it considered “genetic structure” to have a relatively narrow definition … [which] would centre on the expression of *nuclear genes* that result in *heritable characteristics*’.^63^ Second, it decided that ‘genetic structure’ should be understood with reference to a lay person’s understanding of ‘genetic’, so that alteration thereto ‘would involve *alteration to the genes or the genome* and the *resulting heritable characteristics*’.^64^ Third, the Committee reasoned that ‘what might be considered a narrow definition … is aligned with the purposive intent of … the Act’,^65^ observing (with reference to the White Paper), that Parliament had been concerned ‘to restrict techniques which would allow the *artificial creation* of human beings with certain *pre-determined characteristics* through *modification* of an early embryo’s genetic structure’; it also stressed that it thought that the ‘overall’ concern of Parliament was to prohibit ‘*selecting characteristics*, or ensuring a *predisposition* as to *certain characteristics*’.^66^

The Committee’s reasoning relies on a distinction between the nuclear and mitochondrial genomes, supported by scientific and lay opinion. It also highlights a Parliamentary concern with ‘artificial creation’ through ‘modification’ aimed at selecting or shaping specific characteristics. The US IOM report likewise emphasizes the nuclear/mitochondrial distinction and appeals to the ‘public understanding’ of ‘genetic’, observing that ‘[w]hile mtDNA plays a central role in genetic ancestry, traits that are carried in nDNA are those that in the public understanding constitute the core of genetic relatedness in terms of *physical and behavioral characteristics* as well as most forms of *disease*’.^67^

A second aspect of the HFEA Appeal Committee’s reasoning focused, not on the ‘target’ of alteration, but on the ‘manner’ of it. The Committee reasoned that:

[R]emoving the pronuclei from the zygote does not cause the genetic structure to be altered, nor does the depositing of the pronuclei in the cytoplasm of an enucleated egg … [and that] this does not change the genetic structure of the new cell because the nuclear material overrides any DNA in the mitochondrial DNA.^68^

It also considered the meaning of the ‘extended’ phrase ‘altering the genetic structure of any cell while it forms part of an embryo’, reasoning that, while PNT would result in a change to the ‘genetic *constitution or composition*’ of an embryo, it would not result in any change to its ‘genetic *structure*’, referring to its earlier discussion of the latter term.^69^

Subsequently, the question of the way in which MRTs involve changes to embryos or eggs became the second, and minor, theme in the legal and policy debate, to which we now turn.

### B. ‘Replacement’ versus ‘Modification’: the Minor Theme

In the run-up to the passage of the 2015 Regulations, MRTs were justified in part by reference to the nature of the *methods* involved, methods distanced from genetic *modification* on the grounds that instead they amount to *replacement* or *donation.*

This distinction was a focus in various pieces of evidence to the HCSTC in late 2014. For instance, Professor Robin Lovell-Badge, a member of the HFEA’s Review Panel, stated ‘you could call [MRTs] *germline modification* – the HFEA used “germline therapy modification”’.^70^ However, he quickly clarified what sort of ‘modification’ he meant, as follows:

You are *not changing specific DNA sequences*, which is generally how I as an experimental biologist would talk about genetic manipulation or modification. You are *swapping an intact mitochondrial DNA genome* which … *happens anyway through natural reproduction.* It is *not* as if you are *engineering* a specific piece of sequence … I do not see it as a form of genetic modification.^71^

This passage stresses replacement of a *whole* genome, rather than the modification of *parts* of one, and invokes the idea that this occurs in nature anyway. Interjecting, Dr Ted Morrow asked ‘[i]f it is germline modification, how can it not be genetic?’.^72^ Professor Lovell-Badge’s response was that ‘[i]t is. We have not hidden from the fact that it is *germline modification*; we have always said that’.^73^ By itself, this is not fully clear and the only reference to ‘germline modification’ in the HFEA Review Panel’s various reports occurs in its third report, in a footnote reference to ‘genome editing’ techniques which, it states, ‘could not be applied to oocytes or early embryos under the HFE Act, because they would constitute *germline modification* that would require a change in primary legislation’.^74^ So, to make sense of Professor Lovell-Badge’s evidence we may need to interpret him as referring to MRTs as ‘germline modification’ in the same way as the CMO (above), namely ‘simply’ in the sense that the intervention ‘passes through’. The point that specific DNA sequences are not changed by mitochondrial replacement was reiterated by Professor Peter Braude, also a member of the HFEA Review Panel, who stressed: ‘You are *not modifying the actual genome* of the mother and father; you are simply *moving* it into another bag.’^75^

Differences between replacement and alteration were stressed elsewhere, for instance by the Wellcome Trust.^76^ Similarly, the Parliamentary Office of Science and Technology (POST) stated that ‘the changes … involve *swapping* one person’s mtDNA for another’s. This is in contrast to techniques for *modifying nDNA* which involve *snipping* gene sequences from one cell and *splicing* them into another’.^77^ This was with reference to the idea that allowing mitochondrial replacement ‘may have little effect on the consensus not to alter germ line nDNA’.^78^ Likewise, an NCoB Briefing Paper noted that with MRTs ‘[n]either the nuclear envelope nor mitochondrial membranes need be disturbed … [i]n contrast, nDNA modification would, at least, require penetration into the nucleus and probably DNA recombination …’.^79^

These statements all distinguish, in various ways, *modifying* or *altering* a genome (the nuclear one) from *replacing* one entire ‘unhealthy’ genome (the mitochondrial one) with another ‘healthy’ one. The use of terms such as ‘transferring’, ‘swapping’, ‘moving’ or ‘replacing’ a whole genome, as opposed to ‘modifying’ or ‘altering’ parts of one, is striking. It is also noteworthy given that, in the amended HFE Act, the legality of PNT in particular has been established as an exception to the prohibition in Schedule 2, paragraph 1(4), which states (in part) that ‘[a] licence under this paragraph cannot authorise *altering the nuclear or mitochondrial DNA* of a cell while it forms part of an embryo, *except for* the purpose of creating something that will by virtue of regulations under section 3ZA(5) be a permitted embryo’.^80^ This is a revision of the provision in the original Act prohibiting the alteration of the ‘genetic structure of any cell while it forms part of an embryo’.^81^ Somewhat confusingly, though understandably given the revised section’s wording, the DH Explanatory Notes refer to the ‘regulation-making power’ as ‘enabl[ing] eggs and/or embryos with *altered mitochondrial DNA* to be classified as ‘permitted’ eggs or embryos …’.^82^ A similar ambiguity can be found in a House of Commons Library ‘Standard Note’ which states that ‘[t]hese techniques … propose a *change to an embryo’s DNA* prior to implantation’, and that ‘[a]s such, they have been the subject of some opposition and controversy.’^83^ Expressed this way, and in the light of the international background discussed earlier, such controversy is perhaps unsurprising.

Given what is actually involved in MRTs, the only way to understand the Act, the Explanatory Notes and the House of Commons Library statement is to interpret the Act as referring to, and permitting as an exception, ‘alteration’ of the genetic *composition* of the egg or embryo, rather than alteration of mitochondrial DNA *per se.* This would be consistent with the HFEA Appeal Committee’s reasoning: that mitochondrial replacement does involve ‘interventions’ in eggs or embryos (through a ‘replacement’ process), ones that change their genetic ‘composition’, but no alteration to or modification of the *mitochondrial DNA itself.* The emphasis on this last point seems motivated by a wish to distance MRTs from modification of the nuclear genome, no doubt in the light of the history of the HFE Act and the international background. Thus, the contrast between the genomes (mitochondrial versus nuclear) is accompanied by an emphasis on the nature of the intervention: that is, on the substitution of whole ‘natural’ elements, rather than ‘engineered’ changes, seen as ‘artificial’.

To some degree, the same move is made by the US IOM, which contrasts ‘[t]he *replacement of whole, intact*, and *naturally occurring mitochondrial genomes*’ with ‘any approach for *modifying nDNA*, which would likely involve *editing* rather than *en bloc replacement* of chromosomes’.^84^ The Committee argues that there is ‘a qualitatively different form of heritable genetic change’^85^ between the two kinds of intervention, with the connection between the nature of the respective genomes and the relevant methods of intervention in relation to either again being very apparent. The IOM report also observes that ‘[w]hile there is *no direct modification or editing of the mtDNA sequence itself*, the *novel combination* of mtDNA from one woman and nDNA from another would not occur in unassisted sexual reproduction or in other ARTs.’^86^

From here on, however, the UK and US part company. Thus, working with the terms of reference and definitions established by the FDA, the report views ‘genetic modification’ as ‘changes to the genetic material within a cell’.^87^ On this basis, the Committee ‘considers MRT to be “*genetic* modification” of the oocyte or zygote.’^88^ Significantly then, MRTs do constitute ‘*genetic* modification’, not because they involve genome editing, but because they entail a ‘novel combination’ of genetic material. Moreover, noting that ‘germline modification’ is defined by the FDA as ‘human inheritable genetic modification’, the Committee also finds that:

MRT results in the *genetic* modification of germ cells, but … it constitutes heritable genetic modification (*germline modification*) *only if used to produce female offspring* because mtDNA is solely maternally inherited, and therefore MRT to produce male offspring would not constitute heritable genetic modification (germline modification).^89^

Hence, in a significant departure from the UK Government and the HFEA Appeal Committee, the IOM finds that MRTs *would* amount to germline *genetic* modification if female offspring were born.

Of course, while the IOM had to work within the framework established by the FDA, this did not include a legislative background that might bar certain kinds of intervention. By contrast, if the UK HFEA Appeal Committee had not held, as a matter of statutory interpretation, that MRTs do *not* involve ‘genetic modification’ (specifically, ‘alteration’), research into PNT could not have been licensed without a change to the original HFE Act. Thus, defining (by implication) ‘germline genetic modification’ to *exclude* interventions relating only to the mitochondrial genome was essential for research to progress at that time. This move undoubtedly influenced the terms of the UK debate. Significantly however, despite differing from the UK Government position that MRTs are not germline genetic modification, the IOM report is nonetheless consistent with the Government’s emphasis on the mitochondrial/nuclear distinction (together with distinctions between the potential scope and purpose of interventions in either case), and on that between ‘wholesale replacement’ and ‘modification’.

Seen as centrally involving *replacement*, MRTs are aligned with genetic *selection* – long established in prenatal diagnosis (PND) and PGD. They are thereby distanced from genetic *modification* and nuclear genome ‘editing’. In this way, and given the aim of disease prevention, children born through MRTs are positioned far from the hypothetical one discussed in the President’s Council on Bioethics Report, *Reproduction and Responsibility*, in 2004, ‘who … *designed* to certain specifications might be viewed as more of an artefact – or more answerable to the will of his or her parents – than a child who is merely *selected* for his or her existing characteristics’.^90^

In the next section, we examine the (supposed) normative significance of the distinctions between the *mitochondrial* and the *nuclear*, and between *replacement/selection* and *modification.*

## 4. Identity

This section focuses on what was earlier shown to be central to the legal and policy debate: the claim that nuclear genetic modification is more ethically problematic than mitochondrial replacement because it is more likely significantly to affect the resultant child’s identity.

### A. Three Senses of ‘Identity’

We start by drawing a distinction between numerical, qualitative, and narrative identity.

*Numerical Identity.* A and B are identical in this sense if and only if A and B are the very same object or person. So, for example, Theresa May is *numerically identical* with the woman who became British Prime Minister in July 2016 and what this means is that ‘they’ (May and the woman who became Prime Minister) are one and the same woman, not merely two similar women.^91^

*Qualitative Identity.* On a strict definition, A and B are ‘qualitatively identical’ if and only if they share *all* of the same properties or qualities. One obvious response to this is to point out that it is rarely, if ever, the case that two distinct objects are *exactly the same*, because there will always be small differences. If we start also to consider objects’ relational properties (how they are related to other objects in the universe) and their spatiotemporal properties (where and when they are) it is tempting to conclude that, for A and B to be qualitatively identical, they must also be numerically identical, a thesis known as the Identity of Indiscernibles.^92^ An alternative then would be to understand qualitative identity more pragmatically as *extreme similarity*; many pairs of so-called ‘identical twins’ would meet this less exacting standard.^93^

Finally, *Narrative Identity, Self-Conception, or ‘Sense of Self’* are psychological phenomena, but ones with potential ethical significance:

Another relevant sense of identity or self is a psychological, not numerical, sense. It consists of the properties or qualities that an individual considers important to who he is, to what kind of person he is, to what properties of himself he identifies with.^94^

### B. Mitochondrial Replacement and Numerical Identity

If numerical identity is used to distinguish nuclear genetic modification from MRTs, the underpinning claim must be that reproductive technologies which alter the nuclear genome (often) result in a numerically different person coming into being, whereas MRTs (typically) do not. However, this argument is flawed. For it is not clear that the distinction between identity-affecting and non-identity-affecting interventions maps onto a clear ethical line. Nor is it obvious that nuclear modification is identity-affecting while MRTs are not. In what follows we look at each of these points in turn.

(i) *Is the Distinction between Identity-affecting and Non-identity-affecting Interventions Ethically Significant?*

If the distinction between identity-affecting and non-identity-affecting interventions is ethically significant, its significance is neither obvious nor straightforward, and identity-affecting interventions are certainly not *always* wrong or problematic. Imagine that Amelia is planning to use Bobby as a sperm donor. Shortly before donation occurs she discovers that he carries a serious genetic condition; his offspring will suffer from painful and life-shortening disease. Amelia therefore uses another sperm donor instead: Callum, who has no known heritable diseases. In this case, Amelia has changed the (numerical) identity of her future child. She was going to have a child with Bobby’s sperm but instead used Callum’s. A different child ensues, one with a much lower risk of genetic disorder. Many would not think of Amelia’s action as wrong, nor that it is made worse by her child’s (numerical) identity being altered.^95^ Furthermore, other similarly identity-affecting decisions, such as postponing reproduction until one has completed a degree, or moved house, or because of possible exposure to the Zika virus, are ubiquitous and generally regarded as prudent and morally permissible. So, even if nuclear genetic modification were identity-affecting, that would not necessarily make it wrong, or worse than MRTs.

There may however be more subtle ethical differences. One is that whereas non-identity-affecting interventions can directly benefit, cure, or harm determinate future individuals, identity-affecting ones cannot. When a reproductive technology is not identity-affecting and yet has some effect on the future person (for example, if it prevents a genetic disorder) that person (once she exists) can look back upon the intervention and see that she has directly benefitted. It is not so clear however that the same can be said of identity-affecting interventions because the alternative to life with a disorder in such cases is non-existence, not life without a disorder.^96^ Similar reasoning might lead one to think that (some) reproductive interventions not affecting identity can be therapeutic, or at least quasi-therapeutic, in the sense that a determinate (future) person is caused not to have a disease. This does not however apply to identity-affecting interventions. These do not cure anyone. Rather, they involve choosing between possible future persons and ‘screening out’ ones with disease. They are *selective reproduction* rather than *therapy.*

Whether this distinction between therapy and selective reproduction is ultimately ethically significant is beyond the scope of this work. For the present, we merely note that it does open up an interesting line of argument which *could* be used to differentiate some reproductive technologies from others: in this case, nuclear genetic modification from MRTs.^97^ As noted earlier, the idea of therapy also plays a justificatory role in legal and policy debates: against the backdrop of the relevant international statements and conventions in which the goal of treatment is at least acknowledged to be a positive one, in the UK the HFEA, the DH and the NCoB all refer to MRTs as treatment or therapy (a position rejected by the US IOM as regards the future child).^98^

Furthermore, concerns about eugenics and human dignity may be thought to engage more powerfully when interventions are selective rather than therapeutic.^99^ This kind of view can be found, for example, in the Charter of Fundamental Rights of the European Union, Article 3 (‘Right to the integrity of the person’) of which states: ‘In the field of medicine and biology, the following must be respected in particular … the prohibition of eugenic practices, in particular those aimed at the selection of persons.’^100^ There are of course many complex questions about what eugenics is and about its normative status.^101^ However, the general point that the selection or deselection of (possible future) persons can be seen as eugenic, since it is effectively an instance of ‘selective breeding’, is one that people on all sides could accept.^102^

(ii) Is Mitochondrial Replacement Therapy or Selective Reproduction?

The argument that MRTs are less ethically problematic than modifying the nuclear genome, because only the latter is seriously identity-affecting, could only work however if (all or most) genetic modifications of nuclei were identity-affecting while (all or most) instances of mitochondrial replacement were not. Is this plausible? There are two reasons for thinking not. First, some practices termed ‘mitochondrial replacement’ may themselves be identity-affecting. Second, it may be possible to modify a cell nucleus without altering the resultant person’s (numerical) identity. Therefore, the distinction between identity-affecting and non-identity-affecting does not line up in a neat or systematic way with the nuclear/mitochondrial distinction.

As noted earlier, Wrigley and others have plausibly argued that, at least in practice, MST (one kind of MRT) will be identity-affecting because it causes a different sperm to be used at fertilisation.^103^ There may also be scenarios in which the *decision to use* PNT (the other kind of MRT) is identity-affecting. Imagine, for example, the choice between having a child through sexual reproduction versus having a child via IVF and PNT. The chances of the very same egg and spermatozoon coming together at fertilisation in both scenarios are miniscule and even the decision to use IVF could mean that a different person comes to exist, because of that decision’s effects on the timing of conception. But this should come as no surprise for, as has been noted, reproductive decisions affecting numerical identity are ubiquitous. So the attempt to defend MRTs by arguing that they do not (while nuclear genetic modification does) alter (numerical) identity fails. For at least some forms of mitochondrial replacement are identity-affecting; indeed, possibly they all are if we take a wider-context view of the matter and consider not just the technique itself but the position of people who are choosing whether to use IVF and MRTs in the first place.^104^

This conclusion is bolstered by the fact that some instances of nuclear genetic modification may not be identity-affecting. The leading examples here are ones in which nuclear genetic modification makes only a trivial difference.^105^ Imagine, for example, that scientists alter the nuclear genome of an embryo with the only consequence being that the resultant person’s fingers are one millimetre longer. Ought we to say that this person is numerically different? No – for two reasons. First, the qualitative change is minor and superficial and does not (*ex hypothesi*) affect important personal characteristics. Second, intuitively we would want to say that an embryo can survive this small degree of change – that the embryo is altered, not destroyed-and-replaced. The embryo can carry on being (numerically) the same embryo as it was before the intervention – just as an adult might carry on being the same person (and the same organism) despite having an organ removed or replaced.^106^

### C. Mitochondrial Replacement and Qualitative Identity

MRTs will undoubtedly affect ‘qualitative identity’:

[M]odification of the mtDNA is likely to change the (qualitative) identity of the future person … a person without a mtDNA disease will have a different life experience, a different biography and perhaps also a different character.^107^

However, given the meaning of ‘qualitative identity’, this is hardly a surprising claim. If ‘qualitative identity’ is defined stringently, as Bredenoord and others do (they use the expression ‘exactly alike’) then pretty much anything we ever do to a person will affect qualitative identity. As such, the claim that modification of the mitochondrial genome affects qualitative identity is almost trivially true. But even if one takes a broader view of qualitative identity and sees it as merely extreme similarity, MRTs will still affect qualitative identity. For the difference between having and not having a mitochondrial disorder is often huge.

So MRTs alter qualitative identity, but this is not surprising. For this reason, we question whether ‘qualitative identity’ is a very helpful expression. It might be clearer and more informative to talk instead just about altering characteristics or properties. The problem with labelling such changes ‘identity-affecting’ is that it encourages people to think that something more is being claimed than is really the case. If all that is being claimed is that the characteristics of the person are affected, then why not just use the language of altering characteristics or properties?

Having noted these concerns about language, what about the substantive ethical issue? Does the fact that MRTs ‘affect qualitative identity’ have any ethical or legal implications?

There is not in general anything morally troubling about altering qualitative identity. What matters, as the NCoB pointed out in 2012, is not so much *whether* it has been altered but *how*:

Many medical treatments and interventions are intended to improve a person’s health (and thus will change their qualitative identity) compared to their identity had the treatment or intervention not been used. The important ethical question is whether changing the person’s qualitative identity is likely to adversely affect them.^108^

Bredenoord and others suggest that one implication of all this is that it is hard to draw an ethical distinction between nuclear and mitochondrial interventions and they are clearly right that there is no categorical difference (as far as identity is concerned) between modifying nuclear DNA and modifying mtDNA; both alter qualitative identity.^109^ However, there may be other subtler differences.

One is this. Nuclear ‘genome editing’ potentially allows people to manipulate *with considerable precision* important personal characteristics, including significant aspects of behaviour, mind, and personality. But this is not true of MRTs. The latter may have a huge bearing on people’s lives – but crucially this impact is not controlled and targeted to anything like the same degree as that of some conceivable forms of nuclear genetic modification.^110^ This may be ethically significant insofar as the ability precisely to control the makeup of future persons connects with longstanding concerns about ‘designing’ babies. Furthermore, such manipulative power could more easily be extended beyond mere disease-avoidance to enhancement. This is *possible* with mitochondrial replacement, but seems much less *probable* because of the relative lack of fine-grained control that MRTs provide.^111^

A further difference between nuclear genome editing and MRTs is that the former has the potential to introduce into future people characteristics that are fully ‘designed’ or ‘artificial’, unlikely ever to occur naturally. However, this is not true of current MRTs, which involve importing a naturally occurring mitochondrial genome ‘lock, stock and barrel’. We might therefore think that while nuclear genome editing is genetic modification, mitochondrial replacement is selective reproduction. We alluded to this distinction during the earlier discussion of the Non-Identity Problem, but here it does different work. The idea is that selective reproduction is liable to be safer and less ethically troubling because it involves merely choosing between gametes, embryos, or mitochondrial genomes, all of which already exist ‘in nature’ and could easily have gone on to play a role in ‘natural’ reproduction in any event.^112^ We saw earlier that this line of argument – and with it the distinction between ‘replacement’ and ‘modification’ – constituted a second theme of the legal and policy debate, both in the UK and the US.

What ethical significance this has is far from clear, although it may have some. One way of understanding it is as an appeal to the intrinsic superiority of ‘the natural’ over ‘the artificial’ but, for reasons that have been extensively rehearsed elsewhere, any such appeal is doomed to fail.^113^ More promisingly, we may understand it as a claim about risk: that *selecting* between naturally occurring materials is less dangerous than *modifying* things at a genetic level. Clearly there is no necessary connection between the selection/modification distinction and levels of risk; we can imagine selection technologies with dire effects, and conversely benign forms of genetic modification. But perhaps there is some contingent link between the two inasmuch as most known forms of selection (choosing between gamete donors, or between embryos, for example) have quite limited and slow effects on the ‘gene pool’, whereas the genetic modification of embryos could have more dramatic effects. So there may be grounds for taking a more cautious approach to nuclear genetic modification, given that the scale and scope of the risks are greater.

### D. Mitochondrial Replacement and Narrative Identity

Identity in this third sense is intuitively more closely bound up with ethical issues than either numerical or qualitative identity. As DeGrazia puts it:

Most people, most of the time, do not think much about numerical identity. People think more often about their narrative identity. This involves an individual’s self-conception: her most central values, implicit autobiography, and identifications with particular people, activities, and roles.^114^

So what ethical issues does this generate and how do these apply when considering interventions (such as MRTs) which impact directly – not on existing people – but only on the eggs and embryos which may go on to *become* people?

Narrative identity is easiest to understand when applied to the core case: existing adults with mental capacity. We start therefore with this and work back to reproduction. If we think about what might be wrong with modifying an existing adult’s narrative identity, three main kinds of worry emerge: that changes take place without *valid consent*; that changes cause or constitute *inauthenticity*; that changes are *harmful.* Only the last of these applies directly to human reproduction.

Concerns about consent cannot apply because the relevant person does not exist yet and so does not have an opinion. In addition, as Gyngell and others note, it is not clear ‘why the consent of future generations should be seen as vital for decisions involving GGE [germline gene editing] but not for other major decisions with long term effects’, such as environmental policy or the decision to allow (or not) the development of new communications technologies.^115^ Such decisions must always be made by reference to something other than the desires, wishes, or consent of the affected person.^116^

Some may argue that we can appeal instead to *hypothetical* consent. On this view, the position of the future person is like that of the existing child in the following scenario:

A parent is concerned to have her beloved child vaccinated against a deadly and/or debilitating disease. As the child unhappily resists, the parent comforts herself (and perhaps even the child) with the thought that later on, if and when he is more mature, more thoughtful, and more adequately informed about health matters, the adult that the child becomes *will endorse* what the parent has done *and will consent* to comparable measures that might be needed to maintain or enhance the immunity this established.^117^

This may be a useful *heuristic* perhaps but, in reproductive cases, appealing to hypothetical consent does not add much to what we would (or should) do in any event: that is, have regard for the welfare of the child.^118^ For what grounds, other than welfare, could there be for thinking that he or she will come to endorse (or not) a decision to use MRTs? The main one must be that, all things considered, MRTs were good/bad for him/her.^119^ Of course things are more complex than that because there are various ways in which parents or society may influence a future child’s beliefs and values, such that s/he is made retrospectively to be ‘pro’ or ‘anti’ MRTs.^120^ But this added complexity just further diminishes the reliability of hypothetical consent and suggests that basing decisions on speculation about the beliefs of possible future people in 15, 20, or 30 years’ time is unwise. It would be better instead to make the best possible estimate of health and welfare, noting that even this is tremendously difficult. So concerns about consent do not engage in the reproductive case.

Much the same goes for inauthenticity. The main concern here is not that people are literally caused to become someone else, but rather that they are changed in ways which are inconsistent with their ‘true selves’ or ‘authentic values’. While there is much to be said about what ‘authenticity’ is, we do not address this here, since there are some general and fundamental reasons why inauthenticity arguments do not engage.^121^ Foremost amongst these is the fact that, when discussing the modification or selection of gametes or early embryos, *there is no true self* and *there are no values* to be altered in ‘inauthentic’ ways. This is not to say that there are no ethical objections to controlling or ‘designing’ future people’s characteristics.^122^ It is not even to deny that embryos have significant moral status. For even those who ascribe significant moral status to embryos will struggle to make sense of their having the sort of ‘true self’ about which there could be authenticity-concerns. Or indeed, if embryos did already have ‘true selves’, how could we know what they were like and so which changes would or would not make them more or less ‘authentic’? We conclude therefore that authenticity arguments do not engage either.^123^

That just leaves the suggestion that some changes may have *harmful* effects on narrative identity. The question of how MRTs will affect future people’s narrative identities is a complex empirical matter with any claims made being highly speculative. That said, and crucially for the concerns of this paper, the case for assuming any kind of systematic connection between – (a) the extent to which future narrative identity will be adversely affected and (b) whether a modification is *nuclear* or merely *mitochondrial* – appears weak. This is because, as noted earlier, one can imagine relatively trivial forms of nuclear genetic modification, or ones which precisely target a specific disease, *not* having a significant effect on narrative identity. In such cases, the resultant person either may not care at all about their having been ‘modified’ or may understand this as on a par with childhood vaccination. Conversely, being one of the first MRT babies in the world could have a psychological impact on self-conception that is just as significant as being one of the first ‘genome-edited’ children.

In a recent paper on this topic, Jackie Leach Scully asks how MRTs might impact upon narrative identity. She discusses various challenges for MRT children. As a new and unusual social group, they may lack a clear origin-narrative, or their sense of self may be adversely affected by media coverage.^124^ Another factor, one shared with ‘regular’ donor-conceived children, but not interestingly with ‘genetically modified children’, is the existence of the donor:

Perhaps the child’s emerging sense of self could in some way become confused through knowing that a third person was involved in their conception, unlike their peers; children might not be able to understand why the mitochondrial donor is not included in family events and communications; tensions may develop, if the child wants more information about the mitochondrial donor than parents are able or willing to provide.^125^

For these reasons, MRTs are not necessarily in a better position than nuclear genetic modification. Yet neither is the opposite something that we can take for granted because one can also imagine specific narrative identity problems emerging in the case of ‘genetically modified children’: fears about them being ‘designed’, ‘artificial’, or even ‘polluting the gene pool’. So it is possible to imagine problems regarding narrative identity for *both* MRTs *and* nuclear genetic modification. This suggests that there is as yet (and pending further empirical studies on MRT children) no strong narrative identity argument for treating MRTs more favourably than nuclear genetic modification.

### E. Conclusions

Are MRTs less troubling than interventions altering the nuclear genome, because they influence to a lesser extent the identities of future people?

Regarding numerical identity, no clear difference between MRTs and nuclear genome modification was found. Each has the capacity to be identity-affecting, depending on the context, extent, and nature of the procedure; conversely, each could conceivably be used in ways that do not alter numerical identity.

Something similar goes for qualitative identity; both mitochondrial and nuclear interventions would affect this. We did however find that nuclear genome editing may be different from MRTs in some relevant ways. One is that, compared to MRTs, nuclear genome editing provides an ability to manipulate *with considerable precision* important characteristics. Another is that nuclear genome editing has greater potential to introduce characteristics that are fully ‘designed’ or ‘artificial’, and unlikely to occur naturally. These differences might serve partially to justify the suggestion that nuclear genome editing is more dangerous than MRTs and that therefore a more precautionary approach towards nuclear genetic modification is warranted. Nevertheless, two caveats must be stressed. First, it is only a *pragmatic* argument, not a categorical difference between nuclear and mitochondrial. Second, whether this argument should be classified as a concern about *identity* is doubtful. Really the worry here is about the technology generating adverse outcomes and about its going *beyond therapy* towards enhancement and ‘designer babies’.^126^ Neither of these objections need appeal to the notion of identity.

Finally, we concluded that, regarding narrative identity, there is no clear difference between the mitochondrial and nuclear genomes. Interventions in either may or may not be problematic.

## 5. Conclusions

This paper has considered the uses and meanings of the term ‘germline genetic modification’ in the UK and US legal and policy debates and has evaluated the underlying ethical concerns about ‘identity’.

Its analysis has shown that the broadly prohibitive international position regarding germline genetic modification is best understood as relating to the *nuclear* genome and that the concerns in this context relate principally to worries regarding ‘artificial’ changes to ‘identity’, as well as about ‘dignity’, consent, human enhancement, and safety. A central move in the UK and US debates has been to demarcate a clear boundary between the *mitochondrial* genome and the *nuclear* genome and to emphasize the latter’s more significant role in determining personal characteristics and traits. Similarly, *replacing* one mitochondrial genome with another ‘naturally occurring’, ‘donated’ one has been contrasted with, and presented as more innocuous than, ‘altering’, ‘editing’, or ‘modifying’ the nuclear genome (again for reasons, in part, to do with these interventions’ potential to determine identity).

The question of how much normative significance these distinctions have, therefore, is important both to MRTs as a development in reproductive medicine and to the now current question of whether modifications of the *nuclear* genome in reproduction should be permitted. Our analysis has shown that, as far as identity is concerned, MRTs and nuclear genome ‘editing’ are not as different as has been supposed. It does not follow from this that they should be treated alike, since there may be other reasons that justify different legal and policy stances; these include the latter’s greater capacity for non-therapeutic application and its greater potential to effect rapid and radical change in the human ‘gene pool’. It does however mean that one of the central reasons offered for treating MRTs more permissively than nuclear genetic modification, and for not regarding MRTs as ‘germline genetic modification’, is in doubt. The concept of identity cannot, by itself, do the work thus far assigned to it, explicitly or otherwise, in policy debates, international statements and conventions and legal positions.

## Footnotes

^1^ The Human Fertilisation and Embryology (Mitochondrial Donation) Regulations 2015 (the Regulations); entry into force 29 October 2015; foreshadowed in the 2008 revisions to the Human Fertilisation and Embryology (HFE) Act 1990, as discussed below. Approximately 1 in 6,500 children are born each year in the UK with ‘a serious mitochondrial DNA disorder’, often resulting in ‘the premature death of children, painful debilitating and disabling suffering, long-term ill-health and low quality of life’. DH, *Mitochondrial Donation: Government Response to the Consultation on Draft Regulations to Permit the Use of New Treatment Techniques to Prevent the Transmission of a Serious Mitochondrial Disease from Mother to Child* (July, 2014) 5.

^2^ The Human Fertilisation and Embryology Authority (HFEA) itself observed that ‘[i]f mitochondria replacement were to be made available for treatment in the UK, it would be the first time that *modified embryos* were used to make a child. The resulting child will have inherited nuclear DNA from its parents and mitochondrial DNA from a donor.’ HFEA, *Mitochondria Replacement Consultation: Advice to Government* (March, 2013) para 2.12 (emphasis added).

^3^ Discussed in s 2 below. However a US team of doctors, led by Dr Zhang, carried out an MRT procedure in a New York clinic, without applying for Institutional Review Board (IRB) approval, despite apparently contrary recommendations of the American Medical Association (AMA) regarding international research; they then undertook embryo transfer to the intending mother in an affiliated Mexican clinic. See further Mina Alikani and others, ‘First Birth Following Spindle Transfer for Mitochondrial Replacement Therapy: Hope and Trepidation’ (2017) 34(4) Reproductive BioMedicine Online, 333-336, esp 333 and 335, who state that ‘although the requirements for IRB review and approval … were generally met, the shortcomings … may be considered significant’, 333, with various citations including AMA (2010) 12 AMA J Ethics 190-191. The subsequent birth was announced in the latter half of 2016: *New Scientist*, 27 September 2016, in which the baby was reported as five months’ old: https://www.newscientist.com/article/2107219-exclusive-worlds-first-baby-born-with-new-3-parent-technique, last accessed 31 May 2017. Regarding the legal situation in Mexico, see César Palacios-González, ‘The Mexican Mitochondrial Fiasco’ (2016) 871 Bionews 28 September 2016, http://www.bionews.org.uk/page_707057.asp, last accessed 31 May 2017; César Palacios-González and Medina-Arellano María de Jesús, ‘Mitochondrial Replacement Techniques and Mexico’s Rule of Law: On the Legality of the First Maternal Spindle Transfer Case’ (2017) Journal of Law and the Biosciences, 1-20 doi:10.1093/jlb/lsw065; and Tetsuya Ishii, ‘Mitochondrial Replacement Techniques and Mexico’s Rule of Law: On the Legality of the First Maternal Spindle Transfer Case’ (2017) Journal of Law and the Biosciences, 1-7, doi:10.1093/jlb/lsx015.

^4^ See eg Janet Malek, ‘Understanding Risks and Benefits in Research on Reproductive Genetic Technologies’ (2007) 32(4) J Med Philos 339-358 for discussion of the complexity of the risk/benefit ratio.

^5^ DH (n 1) 15, emphases added. Note the definition of ‘germline’ as ‘[t]he sequence of cells that give rise to sperm or egg cells that will pass genetic information on to a child’, in HFEA Review Panel, *Third Scientific Review of the Safety and Efficacy of Methods to Avoid Mitochondrial Disease through Assisted Conception: 2014 update* (June, 2014) 53.

^6^ Institute of Medicine, National Academies of Sciences, Engineering, and Medicine, *Mitochondrial Replacement Techniques: Ethical, Social, and Policy Considerations* (National Academies Press, 2016) doi: 10.17226/21871. But note that the Report recommended (contrary to the UK position) that MRTs should only be used to create male offspring, until sufficient post-birth information regarding the safety of the techniques has been collected, however long this may take, 13. The section in the federal statute referred to is s 749 of the Consolidated Appropriation Act of 2016, passed in December 2015, which states: ‘None of the funds made available by this Act may be used to notify a sponsor or otherwise acknowledge receipt of a submission for an exemption for investigational use of a drug or biological product under section 505(i) of the Federal Food, Drug, and Cosmetic Act (21 U.S.C. 355(i)) or section 351(a)(3) of the Public Health Service Act (42 U.S.C. 262(a)(3)) in research in which a human embryo is intentionally created or modified to include a heritable genetic modification. Any such submission shall be deemed to have not been received by the Secretary, and the exemption may not go into effect.’ See further Eli Y Adashi and I Glenn Cohen, ‘Mitochondrial Replacement Therapy: Unmade in the USA’, (2017) 317(6) JAMA 574-575, who note that as a result ‘absent an FDA ruling on the safety and efficacy of mitochondrial replacement therapy, clinical application is and remains impermissible’, 574; that ‘[t]he moratorium on mitochondrial replacement therapy constitutes an appropriation-dependent policy rider that has to be annually renewed to remain in effect’, 575; and that: ‘The congressional record is mum on the identity of the sponsor or sponsors of the ban, and the precise motives for crafting it remain equally uncertain. The ban’s enactment was all but guaranteed by the complete absence of discussion before its passage or at any time thereafter, and by its inclusion in a must-pass omnibus appropriation bill.’ Ibid.

^7^ As the IOM report notes, although ‘[m]odification of the human germline … is legal in the United States … several regulatory barriers have effectively prevented it from being carried out in many settings’. Ibid 62. On the complexities of the US position, see ch 4 of the report. Since July 2001, ‘the Center for Biologics Evaluation and Research (CBER) of the US Food and Drug Administration (FDA) [has] exercised its jurisdiction over “cell and gene therapy products” and informed ART clinics of a requirement for an Investigational New Drug (IND) application for “therapy involving the transfer of genetic material by means other than the union of gamete nuclei”’. Alikani and others (n 3) 335. Regarding the MRT conducted in the US (n 3), Dr Zhang’s team ‘shared with RBMO editors a pre-IND review request to the FDA, in which they described their past work and desire to continue to offer MRT to selected patients in the USA. However, bound by the December 2015 statute [above (n 6)], the FDA apparently declined the investigators’ request to meet or consider their application’ (ibid).

^8^ Clustered Regularly Interspaced Short Palindromic Repeats.

^9^ See eg the Council of Europe’s Committee on Bioethics, ‘Statement on Genome Editing Technologies’, 2 December 2015, DH-BIO/INF (2015) 13, 2, in which it suggests that the issues raised by CRISPR/Cas-9 could be examined with reference to the principles in the Oviedo Convention, discussed below, and also signals its intention to examine the issues itself; Nuffield Council on Bioethics (NCoB), *Genome Editing: An Ethical Review* (2016), especially s 4 regarding somatic and reproductive uses in humans; and, in particular, the NCoB’s establishment of a new Working Party on ‘genome editing and human reproduction’: http://nuffieldbioethics.org/project/genome-editing/working-party/, last accessed 31 May 2017. For the NCoB’s work on MRTs, see its *Novel Techniques for the Prevention of Mitochondrial DNA Disorders: an Ethical Review* (2012). See further the Hinxton Group: An International Consortium on Stem Cells, Ethics and Law, ‘Statement on Genome Editing Technologies and Human Germline Genetic Modification’ (2015); National Academies of Sciences, Engineering, and Medicine, ‘International Summit on Gene Editing: A Global Discussion’, 1-3 December 2015 (National Academies Press, 2015) doi: 10.17226/21913, which ‘call[ed] upon the national academies that co-hosted the summit – the U.S. National Academy of Sciences and U.S. National Academy of Medicine; the Royal Society; and the Chinese Academy of Sciences – to take the lead in creating an ongoing international forum to discuss potential clinical uses of gene editing; help inform decisions by national policymakers and others; formulate recommendations and guidelines; and promote coordination among nations’, 7; and National Academies of Sciences, Engineering, and Medicine, *Human Genome Editing: Science, Ethics, and Governance* (National Academies Press, 2017) doi: 10.17226/24623.

^10^ *R v Secretary of State for Health, ex parte Bruno Quintavalle (on behalf of Pro-Life Alliance)*, in which the House of Lords adopted a ‘purposive’ interpretation of the Act to enable it to encompass embryos created by cell nuclear transfer (CNR). For discussion, see further Roger Brownsword, ‘Regulating Human Genetics: New Dilemmas for a New Millennium’ (2004) 12 Med LR 14-39.

^11^ HFE Act 1990 (as amended) s 1(1)(b).

^12^ Ibid s 3ZA.

^13^ Ibid s 3ZA(5).

^14^ DH, *Mitochondrial Donation: A Consultation on Draft Regulations to Permit the Use of New Treatment Techniques to Prevent the Transmission of a Serious Mitochondrial Disease from Mother to Child* (February, 2014) 5, emphases in original. The former technique was used in the first MRT birth (n 3). J Zhang and others, ‘First Live Birth Using Human Oocytes Reconstituted by Spindle Nuclear Transfer for Mitochondrial DNA Mutation Causing Leigh Syndrome’, http://www.fertstert.org/article/S0015-0282(16)62670-5/abstract, last accessed 31 May 2017; Mina Alikani and others (n 3); John Zhang and others, ‘Live Birth Derived from Oocyte Spindle Transfer to Prevent Mitochondrial Disease’, (2017) 34(4) Reproductive Biomedicine Online 361-368. The HFEA has now granted the first UK licence to conduct MRT (PNT). HFEA, ‘Licence Committee – Minutes: Centre 0017 (Newcastle Fertility at Life), Variation of Licensed Activities to include Mitochondria Pronuclear Transfer (PNT)’, 9 March 2017.

^15^ The Regulations (n 1) Part 2, paras 3, 4 and 5 regarding eggs, and 6, 7 and 8 regarding embryos. The circumstances concern the ‘particular risk’ of an egg or embryo having ‘mitochondrial abnormalities caused by mitochondrial DNA’, coupled with a ‘significant risk that a person with those abnormalities will have or develop serious mitochondrial disease’.

^16^ Ibid Part 2, paras 3(c) and 6(c) respectively.

^17^ On aspects of the history of the debate, including the main types of arguments that have been used in opposition, see eg Burke K Zimmerman, ‘Human Germ-Line Therapy: The Case for its Development and Use’ (1991) 16 J Med Philos 593-612, 604ff, who refers to ‘germ-line intervention’ being a ‘loaded issue’ in 1991, 604. See also Andrea L Bonnickson, ‘The Politics of Germline Therapy’ (1998) 19 Nature Genetics 10-11.

^18^ HC Deb 1 September 2014, col 112.

^19^ Rec 934 (1982) (emphasis added).

^20^ Indeed, two authors wryly observed ‘[w]e fail to see the “implication”’. R Munson and LH Davies, ‘Germ-Line Gene Therapy and the Medical Imperative’ (1992) 2(2) Kennedy Inst Ethics J 137-158, 142. Article 2(1) of the ECHR, first sentence, states: ‘Everyone’s right to life shall be protected by law.’ Article 3 states: ‘No one shall be subjected to torture or to inhuman or degrading treatment or punishment.’

^21^ Rec 934 (n 19), emphases added.

^22^ IOM (n 6) 28.

^23^ For example, the HFEA (n 2) included multiple references to MRTs as ‘treatment’, eg paras 1.9, 1.18, 1.19, 2.3, 2.12. Similarly, the DH (n 14), 5, introduced MRTs as ‘newly developed treatment techniques to prevent the transfer of a serious mitochondrial disease from a mother to her child’, and made further reference to MRTs as such. It also noted that MRTs ‘would be a form of … germ line gene *therapy* as recognised by reports produced by the HFEA and … [NCoB]’, para 1.27 (emphasis added).

^24^ HFE Act 1990 (as amended) s 11.

^25^ Anthony Wrigley, Stephen Wilkinson and John B Appleby, ‘Mitochondrial Replacement: Ethics and Identity’ (2015) 29 Bioethics 631-638, 638.

^26^ Ibid. Some authors (such as Liao and Rulli) go further and suggest both PNT and MST are selection, rather than therapy. S Matthew Liao, ‘Do Mitochondrial Replacement Techniques Affect Qualitative or Numerical Identity?’ (2017) Bioethics 20-26; Tina Rulli, ‘What Is the Value of Three‐Parent IVF?’ (2016) 46 Hastings Cent Rep 38-47.

^27^ *Regina (Quintavalle) v Human Fertilisation and Embryology Authority (Secretary of State for Health intervening)* [2005] 2 AC 561, in which the House of Lords held that the HFEA could license HLA-typing by PGD to enable a child to be born who would be a tissue match for a sibling with beta thalassaemia major.

^28^ HFE Act 1990 (as amended), Sched 2, para 1(1)(b).

^29^ Ibid s 2(1) (emphasis added).

^30^ Above (n 23).

^31^ IOM (n 6) 6. Of course, this accords with the legal sense of the term ‘treatment services’ in the UK, and the situation of MRTs within these. This finding is a central plank of the report’s ethical analysis, which identifies the welfare of the future child, and minimising risk to that child, as the ‘primary value’. Ibid 116.

^32^ Ibid 87.

^33^ President’s Commission, *Splicing Life: A Report on the Social and Ethical Issues of Genetic Engineering with Human Beings* (President’s Commission for the Study of Ethical Problems in Medicine and Biomedical and Behavioral Research, 1982) 48.

^34^ Ibid 78.

^35^ National Human Genome Research Institute, *International Human Genome Sequencing Consortium Publishes Sequence and Analysis of the Human Genome* (12 February 2001).

^36^ In a 2002 report the President’s Council on Bioethics stated that ‘[t]he notion of cloning raises issues about identity and individuality’. President’s Council, *Human Cloning and Human Dignity: An Ethical Enquiry* (PCBE, 2002). For further discussion on individuality and uniqueness, see Dan Brock, ‘Human Cloning and Our Sense of Self’ (2002) Science 314-316. These developments had reverberations even in EctHR jurisprudence not obviously in point. Eg in *Vo v France*, 53924/00 [2004] 2 FCR 577, concerning the applicability of Article 2 (the right to life) to the fetus, the dissenting judges expressed concerns regarding ‘genetic engineering’ (Dissenting Opinion of Judge Ress, para 5), ‘genetic manipulation and the risk that scientific results will be used for a purpose that undermines the dignity and identity of the human being’ (Dissenting Opinion of Judge Mularoni, joined by Judge Strážnická (no paragraph numbers).

^37^ UNESCO, *Report of the IBC on Preimplantation Genetic Diagnosis and Germline Intervention* (2003), Annex 2; the UK reference therein is to the HFE Act 1990 Sched 2, para 3(4), discussed below. See further BioPolicyWiki, ‘Inheritable Genetic Modification’ (2014) available at http://biopolicywiki.org/index.php?title=Inheritable_genetic_modification, last accessed 31 May 2017.

^38^ Robert Andorno, ‘Biomedicine and International Human Rights Law: in Search of a Global Consensus’, (2002) 80(12) Bull World Health Organ 959-963, 961. Andorno notes that, at the time of writing, he is a Member of the UNESCO International Bioethics Committee.

^39^ The NCoB has suggested that an important question in relation to genome editing is who the ‘public’ is in ‘public interest’ and whether, for instance, ‘the content of … [the] interest [in genome editing can] … be determined independently for a given political community or … [whether] it [is] coextensive with the scope of universal human rights’. NCoB (2016) (n 9) para 4.42.

^40^ Emphases added.

^41^ Emphases added.

^42^ DH (n 14) para 1.29 (emphasis added). Contextualising the UNESCO declaration, the UK Government elsewhere observed that ‘UNESCO declarations are statements of principles or a common standard of achievement, which are not signed or ratified and are not legally binding.’ DH (n 1) 16.

^43^ In the UK, see the HFEA’s advice regarding long-term studies of children born following the use of MRTs, HFEA (n 2) para 6.33; in the US, see the IOM (n 6) 12, Rec 3. On the empowering role of the notion of dignity in human rights analyses, as opposed to its constraining role for ‘dignitarians’, see Roger Brownsword, ‘Regulating Human Enhancement: Things Can Only Get Better?’ (2009) 1(1) Law, Innovation and Technology 125-152, 129.

^44^ UNESCO, IBC, ‘Report of the IBC on Updating its Reflection on the Human Genome and Human Rights’ 2 October 2015, SHS/YES/IBC-22/15/2 REV.2, para 118, emphasis in original. The specific reference to CRISPR/Cas-9 (in para. 102) refers to developments in China announced in April 2015. The references to the NCoB are to its 2012 report (n 9).

^45^ IOM (n 6) 93.

^46^ Convention for the Protection of Human Rights and Dignity of the Human Being with regard to the Application of Biology and Medicine, ETS No 164, Oviedo, 4 April 1997. The description of the Convention by the Council is at: http://www.coe.int/en/web/bioethics/oviedo-convention, last accessed 31 May 2017.

^47^ Emphases added.

^48^ The idea of *intentional* interferences is discussed in John EJ Rasko, Gabrielle M O’Sullivan and Rachel A Ankeny, ‘Is Inheritable Genetic Modification the New Dividing Line?’ in John EJ Rasko, Gabrielle M O’Sullivan and Rachel A Ankeny (eds), *The Ethics of Inheritable Genetic Modification* (Cambridge University Press, 2006) 1-15, 5.

^49^ See eg North East England Stem Cell Institute (NEESCI), ‘Briefing Paper on the Need to Protect the Future Possibility of Treating Mitochondrial Disease and Other Conditions by a Procedure that Involves Mitochondrial Transplantation’ (May, 2008) 4.

^50^ Council of Europe (n 9) 13, 2.

^51^ For criticism see eg Annelien L Bredenoord, Rieke van der Graaf and Johannes J M van Delden, ‘Towards a “Post-human Dignity Area” in Evaluating Emergent Enhancement Technologies’ (2010) 10(7) AJOB 55-57, 56.

^52^ Andorno (n 38) 960.

^53^ John A Robertson, ‘Oocyte Cytoplasm Transfers and the Ethics of Germ-Line Intervention’ (1998) 26 J Law Med Ethics 211-220, 216.

^54^ HC Deb 3 February 2015, col 162. Her answer is somewhat unclear: ‘It has to be said that there is no universally agreed definition of genetic modification, but for the purposes of these regulations, we have used a working definition and it involves not altering the nuclear DNA.’

^55^ DH (n 1) 15 (emphases added). The Government’s approach had been the subject of some criticism in July 2014, including from scientists such as Lord Winston and Dr Ted Morrow. See http://www.independent.co.uk/news/science/exclusive-scientists-accuse-government-of-dishonesty-over-gm-babies-in-its-regulation-of-new-ivf-technique-9631807.html, last accessed 31 May 2017.

^56^ House of Commons Science and Technology Committee (HCSTC), *Oral Evidence: Mitochondrial Donation*, HC 730, 22 October 2014, 25 (emphases added).

^57^ DH (n 14) para 1.5 (emphases added). See eg NEESCI (n 49) 4.

^58^ Ibid para 1.27 (emphases added).

^59^ HFEA, ‘Mitochondrial DNA Disorders – Is There a Way to Prevent Transmission? Summary of How the HFEA Made its Decision to Licence this Project of Research’ (2005) RO153.

^60^ Ibid para 13.

^61^ HFE Act 1990, Sched 2, para 3(4), emphasis added.

^62^ HFEA (n 59) para 14.

^63^ Ibid para 16 (emphases added).

^64^ Ibid para 17 (emphases added).

^65^ Ibid para 17.

^66^ Ibid para 18 (emphases added).

^67^ IOM (n 6) 107 (emphases added).

^68^ HFEA (n 59) para 20.

^69^ HFEA (n 59) para 22 (emphases in original).

^70^ HCSTC (n 56) 13 (emphasis added).

^71^ Ibid (emphases added).

^72^ Ibid.

^73^ Ibid (emphasis added).

^74^ HFEA Review Panel (n 5) 37, n 41 (emphasis added).

^75^ HCSTC (n 56) 14 (emphases added).

^76^ The Wellcome Trust emphasized that mitochondrial replacement ‘allows for *unaltered* nuclear DNA to be transferred to an egg or embryo that has *unaltered* healthy mitochondria …. These techniques therefore only *replace*, rather than *alter*, a small number of unhealthy genes in the “battery pack” of the cells with healthy ones.’ Wellcome Trust Written Evidence (MIT0008), HCSTC (n 56) para 4 (emphases added),

^77^ POST, ‘Preventing Mitochondrial Disease’ (2013) 431 (March) Postnote 4 (emphases added).

^78^ Ibid, citing the NCoB report’s reference to a ‘distinct material boundary’ between the nuclear and mitochondrial genomes: ‘The fact that there is a distinct material boundary between mitochondrial and nuclear genes allows regulators to establish an equally clear legal distinction between modifications to the different genomes, thereby forming a practical barrier to the threat of “slippery slope” arguments.’ NCoB 2012 (n 9) para 4.65. In turn, the NCoB had referred to a number of relevant statements, including the NEESCI (n 49), 4, which states: ‘Germline gene therapy is a term used for modifying genes in the *nuclear* genome at the beginning of development with the *intention of changing the organism in a specific way and for potentially transmitting this change to subsequent progeny* …. Replacing diseased mitochondria with healthy ones is an inherently less complicated procedure. *No genome is being modified. Whole mitochondria are being replaced.*’ (Emphases added.)

^79^ Mark S Frankel and Brent T Hagen, ‘Germline Therapies’ (NCoB, 2011) para 13.

^80^ HFE Act 1990 (as amended), s 3ZA(5), emphases added.

^81^ HFE Act 1990, Sched 2, para 3(4), discussed in text following n 61.

^82^ HFE Act 2008, Explanatory Notes (2008) para 162 (emphasis added).

^83^ House of Commons Library, ‘Mitochondrial Donation’, Standard Note: SN/SC/6833, 30, para 8 (emphasis added); 16, para 5.1.

^84^ IOM (n 6) 107 (emphases added).

^85^ Ibid.

^86^ Ibid 88 (emphases added).

^87^ Ibid.

^88^ Ibid (emphasis added).

^89^ Ibid 6 (emphases added); see further 88-89.

^90^ President’s Council on Bioethics, *Reproduction and Responsibility: The Regulation of New Biotechnologies* (PCBE, 2004) 109 (emphasis in original).

^91^ Aristotle, *Topics*, 103a6-103a38B; John Locke, *An Essay Concerning Human Understanding* (WLC Books, 1690) ch 27.

^92^ Leibniz writes that ‘ … it is never true that two substances are entirely alike, differing only in being two rather than one’. Gottfried von Leibniz, *Discourse on Metaphysics* (St Martin’s Press, 1988) s 9.

^93^ As Bredenoord and others put it: ‘Identical twins may be qualitatively identical, meaning they are exactly alike. Numerically, though, they are different: they are two different persons.’ Annelien L Bredenoord and others, ‘Ethics of Modifying the Mitochondrial Genome’ (2011) 37 J Med Ethics 97-100, 98.

^94^ We do not attempt here to separate out Narrative Identity, Self-Conception, or ‘Sense of Self’. It may be that these are subtly different things but, for the present, we are grouping them together because they seem to be doing essentially the same work in the mitochondrial replacement debate. Dan Brock, ‘Human Cloning and our Sense of Self’ (2002) 296 Science 314-316.

^95^ Some would go further and say that her decision is morally praiseworthy, or even obligatory, because avoiding disease and suffering is a good thing. Eg Julian Savulescu, ‘Procreative Beneficence: Why We Should Select the Best Children’ (2001) 15 Bioethics 413-426; Stephen Wilkinson, *Choosing Tomorrow’s Children: the Ethics of Selective Reproduction* (OUP, 2010) 90-96.

^96^ Jonathan Glover, *Causing Death and Saving Lives* (Penguin, 1977) 51-53; Derek Parfit, *Reasons and Persons* (Oxford University Press, 1986) 358.

^97^ For an interesting discussion see eg Pilar Ossario, ‘Inheritable Genetic Modifications: Do We Owe Them to Our Children?’ in Audrey Chapman and Mark Frankel (eds), *Designing Our Descendants: The Promises and Perils of Genetic Modifications* (Johns Hopkins University Press, 2003) 253-271.

^98^ Some of the academic debate has also coalesced around this subtopic of whether mitochondrial replacement is therapy or reproductive (de)selection. Rulli (criticizing Arthur Caplan) makes the point as follows: ‘ … Caplan states that the procedure “is not without its risks, but it’s treating a disease. These little embryos, these are people born with a disease,” he says. “[T]hey can’t make power. You’re giving them a new battery. That’s a therapy. I think that’s a humane ethical thing to do.” This is an inaccurate picture of the procedure. The technology would not treat a child born with mitochondrial disease; the whole point of the technology is that a child would be born without this disease at all.’ Rulli (n 26) 41.

^99^ Callum MacKellar, ‘Should Persons Affected by Mitochondrial Disorders Not be Brought into Existence?’ (2014) 736 Bionews, http://www.bionews.org.uk/page_385343.asp, last accessed 31 May 2017; Stephen Wilkinson and Eve Garrard, *Eugenics and the Ethics of Selective Reproduction* (Keele University, 2013) https://www.keele.ac.uk/media/keeleuniversity/ri/risocsci/eugenics2013/Eugenics%20and%20the%20ethics% 20of%20selective%20reproduction%20Low%20Res.pdf, last accessed 31 May 2017.

^100^ 2012/C 326/02.

^101^ Arthur Caplan, Glen McGee and David Magnus, ‘What is Immoral about Eugenics?’ (1999) 319 BMJ 1284-5; Jonathan Glover, *Choosing Children: Genes, Disability and Design* (OUP, 2006); Stephen Wilkinson, ‘“Eugenics Talk” and the Language of Bioethics’ (2008) 34 J Med Ethics 467-471; Wilkinson and Garrard (n 99).

^102^ That said, even curative interventions might be thought of as having eugenic aims if part of their purpose is ‘improving the genes’ of future generations – but then the same can be said of many interventions (medical or otherwise) that affect future generations.

^103^ Wrigley and others (n 25) 635. See also César Palacios-González, ‘Mitochondrial Replacement Techniques: Egg Donation, Genealogy and Eugenics’ (2016) 34 Monash Bioethics Review 37-51.

^104^ Liao (n 26) agrees with us, though for different reasons, and his position is ‘stronger’. He argues that ‘MRTs create a new and numerically distinct child’ because ‘the enucleation process involved in MST and PNT permanently disrupts the cellular or organismic continuity in an egg or zygote’.

^105^ Nicola Williams, ‘Possible Persons and the Problem of Prenatal Harms’ (2013) 17 J Ethics, 355-385; Clark Wolf, ‘Do Future Persons Presently have Alternate Possible Identities?’ in Melinda Roberts and David Wasserman (eds) *Harming Future Persons* (Springer, 2009) 93-114.

^106^ See also Liao (n 26) who disagrees on this point.

^107^ Bredenoord and others (n 93) 99.

^108^ NCoB (2012) (n 9) 53.

^109^ Bredenoord and others (n 93) 99.

^110^ Bredenoord and others suggest that when a child ‘inherit[s] a manipulated genome’ this ‘can be perceived as a violation of its genetic integrity’. Annelien Bredenoord, Guido Pennings and Guido de Wert, ‘Ooplasmic and Nuclear Transfer to Prevent Mitochondrial DNA Disorders: Conceptual and Normative Issues’ (2008) 14 Hum Reprod Update 669-678, 674.

^111^ Human enhancement is a complex and controversial topic and not one on which we can take a view here. See eg Nicolas Agar, *Liberal Eugenics: In Defence of Human Enhancement* (Blackwell, 2004); Nick Bostrom, ‘Human Genetic Enhancements: A Transhumanist Perspective’ (2003) 37 J Value Inq 493-506; Glover (n 101); Jonathan Glover, *What Sort of People Should There Be? Genetic Engineering, Brain Control, and Their Impact on Our Future World* (Penguin, 1984); John Harris, *Enhancing Evolution: The Ethical Case for Making Better People* (Princeton University Press, 2007).

^112^ Christopher Gyngell, Thomas Douglas and Julian Savulescu, ‘The Ethics of Germline Gene Editing’ (2016) J Appl Philos doi:10.1111/japp.12249.

^113^ Julian Baggini, *Making Sense: Philosophy Behind the Headlines* (OUP, 2002) 4.

^114^ David DeGrazia, ‘Enhancement Technologies and Human Identity 1’ (2005) 30 J Med Philos 261-283, 266.

^115^ Gyngell and others (n 112).

^116^ Obviously we must also consider the need, in reproductive cases, to procure valid consent from the prospective parents and any third-party donors, but this is a quite different kind of issue.

^117^ Arthur Kuflik, ‘Hypothetical Consent’ in Franklin Miller and Alan Wertheimer (eds), *The Ethics of Consent: Theory and Practice* (OUP, 2010) 131-162, 132.

^118^ Our approach to hypothetical consent in these cases then is like that described by Kuflick, as follows: ‘What relevance hypothetical consent might have is entirely parasitic upon other claims that must be defended more directly in any event. Once these claims are plausibly in view, the appeal to hypothetical consent is superfluous’. Kuflik (n 117) 134.

^119^ In cases where *numerical* identity is affected, things are more complex. But still we may ask: will the intervention result in a child who is ‘better off’ than the alternative (possible future) child would have been? This in in effect just an ‘impersonal’ version of the very same welfare question.

^120^ Jackie Leach Scully, ‘A Mitochondrial Story: Mitochondrial Replacement, Identity and Narrative’ (2017) Bioethics 37-45.

^121^ Carl Elliott, *A Philosophical Disease: Bioethics, Culture and Identity* (Routledge, 1999); Alessandro Ferrara, *Reflective Authenticity* (Routledge, 1998); Charles Guignon, *On Being Authentic* (Routledge, 2004); Charles Taylor, *Sources of the Self: The Making of the Modern Identity* (Cambridge University Press, 1989); Somogy Varga, *Authenticity as an Ethical Ideal* (Routledge, 2011).

^122^ See eg Michael Sandel, *The Case against Perfection* (Harvard University Press, 2007).

^123^ For the reasons given here, inauthenticity-arguments may have more traction when applied to therapies (genetic or otherwise) for existing people (especially those who have lived long enough to develop an ‘authentic’ self) than they do in the reproductive context. See eg Felicitas Kraemer, ‘Authenticity or Autonomy? When Deep Brain Stimulation Causes a Dilemma’ (2013) 39 J Med Ethics 757-760; Felicitas Kraemer, ‘Authenticity Anyone? The Enhancement of Emotions via Neuro-psychopharmacology’ (2011) 4 Neuroethics 51-64.

^124^ Scully (n 120) 40.

^125^ Ibid.

^126^ In its 2017 report (n 9) the National Academies of Sciences, Engineering, and Medicine states, in Recommendation 5.1: ‘Clinical trials using heritable germline genome editing should be permitted only within a robust and effective regulatory framework that encompasses: the absence of reasonable alternatives; restriction to *preventing a serious disease or condition* …’ amongst a list of other criteria (emphasis added). Recommendation 6.1 states: ‘Regulatory agencies should not *at this time* authorize clinical trials of somatic or germline genome editing for purposes other than treatment or prevention of disease or disability.’ (Emphasis added.) The report observes, 147: ‘Significant scientific progress will be necessary before any genome-editing intervention for *indications other than the treatment or prevention of disease or disability* can satisfy the risk/benefit standards for initiating a clinical trial.’ (Ibid, emphasis added.) It urges ‘robust public discussion’ regarding non-therapeutic purposes, ibid.

